# Embedding formal and experiential public and patient involvement training in a structured PhD programme: process and impact evaluation

**DOI:** 10.1186/s40900-023-00516-4

**Published:** 2023-11-24

**Authors:** Maria Pierce, Louise Foley, Bridget Kiely, Aisling Croke, James Larkin, Susan M. Smith, Barbara Clyne, Edel Murphy

**Affiliations:** 1 School of Social Policy, Social Work and Social Justice, University College Dublin, Dublin, Ireland; 2 School of Allied Health, University of Limerick, Limerick, Ireland; 3 Department of General Practice, RCSI University of Medicine and Health Sciences, Dublin, Ireland; 4 Discipline of Public Health and Primary Care, Trinity College Dublin, Dublin, Ireland; 5 PPI Ignite Network, University of Galway, Galway, Ireland

**Keywords:** Public and patient involvement, Multimorbidity, Doctoral research, Evaluation

## Abstract

**Background:**

Incorporating Public and Patient Involvement (PPI) into doctoral research is valued by PhD funders and scholars. Providing early career researchers with appropriate training to develop skills to conduct meaningful PPI involvement is important. The Health Research Board (HRB) Collaborative Doctoral Award in MultiMorbidity programme (CDA-MM) embedded formal PPI training in its structured education. The four participating PhD scholars established a PPI panel comprising people living with two or more chronic conditions, presenting an opportunity for experiential PPI training. This study aimed to evaluate the process and impact of embedding PPI training in a structured PhD programme.

**Methods:**

This study was a longitudinal mixed-methods evaluation, conducted over 24 months (June 2020 to June 2022). A process evaluation provided an understanding of how PPI was embedded and explored the experiences of key stakeholders involved. An impact evaluation assessed the impact of embedding PPI training in the programme. Participants included PhD scholars, PPI contributors and PhD supervisors. The data collection and analysis was led by an independent researcher not aligned with the CDA-MM. Data collection methods included five focus groups, individual interviews (n = 6), an impact log, activity logs and group reflections. Qualitative data were analysed using thematic and content analysis and quantitative data analysed using descriptive statistics.

**Results:**

Embedding formal and experiential PPI training in a structured PhD programme is feasible. Both approaches to training are fundamental to building PPI capacity. Involvement of an experienced and knowledgeable PPI lead throughout is perceived as critical. The PPI panel approach offered a good example of embedded consultation and worked well in a structured PhD programme, providing PhD scholars with ample opportunities for learning about PPI and its implementation. For PPI contributors, culture was the most important indicator of quality and was positively evaluated. Key roles for PhD supervisors were identified. Embedding formal and experiential PPI training impacted positively on many different aspects of individual PhD research projects and on PhD scholars as researchers. There were positive impacts for PPI contributors and PhD supervisors.

**Conclusions:**

Embedding formal and experiential PPI training in a structured PhD programme is a novel approach. The evaluation has identified a number of lessons that can inform future doctoral programmes seeking to embed formal and experiential PPI training.

**Supplementary Information:**

The online version contains supplementary material available at 10.1186/s40900-023-00516-4.

## Abstract (plain language summary)

Four PhD scholars participated in the CDA-MM. They received training and support from a PPI lead on how to conduct PPI in research. They established a PPI panel of people with two or more ongoing health conditions, to enable PhD scholars to get input from PPI contributors and learn how to do PPI well. An evaluation study was conducted to explore how the PhD scholars conducted PPI, how well it worked, the difference it made and to identify messages for PhD scholars wishing to involve PPI contributors. For the evaluation, the PPI contributors, PhD scholars and PhD supervisors were asked about their experiences and views. For many of the PPI contributors, being part of the CDA-MM PPI panel was their first experience of being involved in PPI. The ongoing support they received from PhD scholars was important. For them, relationships and the way that meetings are conducted matter for doing PPI well. They liked working in small groups and on concrete issues. They found the time they were expected to give was reasonable and within acceptable limits. They preferred in-person meetings. According to PPI contributors, when PPI is done well, it has benefits for the research, particularly ensuring that plain language is used and jargon avoided when researchers communicate with people with two or more ongoing health conditions. PhD scholars benefit from getting the patient perspective and learning how to communicate their research to patients. PPI contributors benefit in many different ways. Some PPI contributors argued that the PPI advisory panel worked so well in the CDA-MM that no changes were needed, whereas others would like to explore different ways of being involved in research.

**Supplementary Information:**

The online version contains supplementary material available at 10.1186/s40900-023-00516-4.

## Background

Public and Patient Involvement (PPI) can be defined as research undertaken with or by patients and the public, as opposed to research undertaken on, for, or about them[1]. Internationally, the importance of PPI has increasingly been recognised [2, 3], driven by policy initiatives, community and patient organisations and academics and practitioners [4]. Increasingly, PPI in research is promoted by funders who link funding to PPI [5], encouraging PPI to become the norm in many jurisdictions [4].

The international evidence shows that PPI is feasible, but evidence on the economic costs of PPI in research is sparse [6]. It shows that drawing on the lived experience of PPI contributors can have positive benefits for health and social care research. It can lead to better outputs [7], and while it can enhance the quality and relevance of studies the evidence base on its impact remains weak [3]. Understanding of the impact of PPI in research is hindered by the poor quality of reporting of PPI, despite the availability of tools, frameworks, guidelines, and critical appraisal checklists for reporting [8]. For PPI contributors, involvement can be beneficial but is also challenging, which brings to the fore the importance of optimising the context and process of involvement [9].

There is evidence that PPI in health research can become tokenistic [10] and concern that inviting patients and the public to ‘tinker at the edges’ [11] undermines the broader aim of PPI to democratise research [12]. International evidence shows that researchers are willing to change their practice, but lack of knowledge, skills and experience can hinder their involvement in PPI [13] and they may be apprehensive about using PPI [14]. Studies have shown that PPI training raises awareness and increases understanding among researchers of the value and relevance of PPI in research. It ensures that they have the necessary skills to embed PPI in the research process [14, 15] and provides the confidence needed to carry out PPI [16]. The cultural context in which researchers operate also has an influence on how researchers implement PPI [13].

The importance of providing early career researchers with appropriate education and training to enhance understanding and skills to conduct meaningful PPI has been recognised [13, 17]. There have been calls to embed PPI as a component of postgraduate education [17, 18] and there is increased awareness of PPI amongst doctoral scholars and their supervisors [19, 20]. Internationally, there is a small but growing number of studies reporting on PPI in doctoral training programmes [18, 20–25]. These studies, in which PhD scholars present their personal reflections, describe different approaches to embedding PPI and demonstrate how it can be incorporated throughout the research process, show that PPI has the potential to positively contribute to the development of both doctoral research project and PhD development. They show that it can be a rewarding experience for PPI contributors, but is not without its challenges. Most recommend greater uptake of PPI in doctoral research but argue that this needs to be adequately supported with training so that PhD scholars can overcome the challenges highlighted [18, 21–23]. However, the incorporation of formal PPI training in doctoral programmes is in its infancy. While examples are emerging of PPI education and training embedded in health-related doctoral programmes, e.g. Structured Population health, Policy and Health-services Research Education (SPHeRE) programme in Ireland, there are very few examples describing how to operationalise PPI in such programmes [19].

PPI is increasingly in evidence in Ireland [26]. In 2017, two national research funders, the Health Research Board (HRB) and the Irish Research Council (IRC), funded the PPI Ignite programme, supporting five universities to embed PPI across their research culture [27]. The PPI Ignite programme, which focused on building PPI capacity amongst researchers as well as patients and the public, has now evolved into the PPI Ignite Network [27]. The PPI Ignite Programme and PPI Ignite Network have been key drivers of the growing interest in PPI amongst researchers in Ireland in recent years. They have led to an increase in PPI training courses and conferences, development of general and disease-specific PPI groups, and growing numbers of partnerships between patient organisations and academic research groups, as demonstrated by the list of events on the PPI Ignite Network website.

### The collaborative doctoral award in multimorbidity programme (CDA-MM)

The Collaborative Doctoral Award in MultiMorbidity programme (CDA-MM) is a HRB funded postgraduate research programme that commenced in Autumn 2018 and includes a cohort of four PhD scholars in primary care, from different disciplinary backgrounds (general practice, health economics, health psychology and pharmacy). CDA-MM is underpinned by the research theme of multimorbidity, defined as the presence of two or more chronic conditions in one individual [28]. Involving patients as partners in multimorbidity research may help to answer complex clinical questions for this population [29]. Some evidence of the positive impact of PPI in multimorbidity research has already been reported [30] It is recognised that involving people living with multimorbidity in research is feasible, and the challenges of identifying PPI contributors who reflect the diversity of people with multimorbidity, including sub-groups with health determinants such as low education, low income and living in rural locations, and of engaging them in a meaningful way can be overcome [31]. PPI has been a core element of the CDA-MM since its inception: during the funding application process, an existing PPI group working on other primary care studies with members of the applicant team contributed to shaping the research questions of the individual PhD studies and the overall aims of the CDA-MM. The CDA-MM curriculum incorporated a year of structured PhD training, designed to include PPI components; and the Steering Group of experienced interdisciplinary multimorbidity researchers leading the CDA-MM was committed to embedding PPI across the doctoral programme.

The CDA-MM was the first PhD programme internationally with a multimorbidity theme and the first in Ireland to embed PPI training within its structured PhD programme. The participating PhD scholars completed formal PPI training, supplementary to a series of general training modules provided through the SPHeRE Programme. Formal PPI training was planned and coordinated by the CDA-MM PPI lead. The training comprised five sessions, delivered by the PPI lead, PPI contributors and other leaders in PPI, communication and public engagement leads, and the evaluation lead:

- PPI in primary care research
- Establishing a PPI panel for the CDA-MM
- Online PPI facilitation skills
- Conducting a PPI evaluation study
- Communicating research findings to the public.

To build on formal PPI training, the PhD scholars established and worked with a PPI panel. This provided them with an opportunity for ongoing experiential PPI learning. Evidence is lacking on the process and impact of embedding PPI training across a doctoral programme. Therefore, this study aimed to evaluate how formal and experiential PPI training were embedded in a structured doctoral programme and the impact of this training on the individual PhD projects and the overall programme. Specifically, the objectives of the study were to:

- Explore the feasibility of embedding formal and experiential PPI training within a doctoral programme.
- Explore the experiences and perspectives of PPI contributors participating in a doctoral programme PPI panel.
- Explore the process and perceived impact of embedding formal and experiential PPI training in the CDA-MM from the perspectives of PhD scholars and PhD supervisors.
- Assess the impact of embedding PPI in the CDA-MM on the design and conduct of the four individual research projects being undertaken by the PhD scholars, as well as on the overall CDA-multimorbidity programme, including training and dissemination activities.

In addition to including PPI panel members in the evaluation as study participants, the study aimed to engage the PPI panel in planning the evaluation study and reviewing evaluation findings.

## Methods

### Study design

A detailed outline of the study design is provided in the protocol [32], which also outlines the roles and responsibilities of the authors/researchers. For reasons related to objectivity, credibility and ethical considerations, an external researcher (MP) not otherwise associated with the CDA-MM and not involved in CDA-MM governance led the data collection and data analysis for the evaluation. As outlined in the protocol, this study is a longitudinal, mixed-methods evaluation conducted over a 24-month period during the 48 month-long CDA-MM. The first and second rounds of data collection took place at 24 and 36 months, respectively. A convergent parallel mixed method design was used, whereby the qualitative data and quantitative data were first analysed separately and then the results were compared to see if either supported the other or not [33]. A ‘constant comparison’ approach was adopted to help ensure that the data from all sources was treated as a whole. The evaluation period ended before the formal end of the programme to ensure time for data analysis, engagement with the PPI panel about results, and report and paper writing. This paper reports on the completed study according to the Good Reporting of a Mixed Methods Study (GRAMMS) guidance framework [see Additional file 1] [34]. The evaluation included two key elements: a process evaluation and an impact evaluation.

### Process evaluation

Qualitative methods were used to explore the process of embedding formal and experiential PPI training in the CDA-MM, and to explore the experiences and perspectives of the 20 participants—eight PPI contributors, four PhD scholars and eight PhD supervisors—on embedding PPI in a structured PhD programme. A combination of focus group and individual interviews conducted at specified time points after the establishment of the PPI panel were used to explore the experiences and views of these key stakeholders:

- Two focus groups were conducted with PPI contributors (24 months (n = 8) and 36 months (n = 5)).
- Two focus groups were conducted with PhD supervisors (24 months (n = 8) and 36 months (n = 5)).
- One focus group (24 months) was conducted with four PhD scholars, each of whom also participated in an individual interview (36 months).

Focus groups and individual interviews were semi-structured, guided by topic schedules informed by the existing literature and with input from the PPI panel. The semi-structured approach allowed the researcher to remain flexible and adapt questions in response to participants [35].

### Impact evaluation

A mix of qualitative and quantitative methods were used to assess the perceived impact of PPI on the PhD projects, PhD scholars’ learning and development, and on PPI contributors, and to assess PhD scholars’ time contributed to PPI activities. Data were gathered from:

- *Two sample activity logs*: To quantitatively assess the time involved in organising, planning, and conducting PPI panel meetings for the CDA-MM, the PhD scholars completed a detailed activity log for two PPI panel meetings, one during the first 12 months, and another during the final 12 months of the study [see Additional file 2 for Activity Log template]. Data was recorded in a shared excel sheet and summarised using descriptive statistics.
- *Impact logs completed by PhD scholars*: To assess the impact of embedding PPI in the CDA-MM on the design and conduct of the four individual PhD research projects, impact logs were completed by PhD scholars after each PPI meeting. The impact logs used a common template developed for the purpose of assessing impact [see Additional file 3]. Data were recorded in a shared excel sheet. In February 2022, the PhD scholars finalised their impact log entries and data were analysed. Qualitative data from the impact logs were analysed thematically to summarise the PPI activities in which the PhD scholars involved PPI panel members and the PhD scholars’ perceived impact of PPI activities on individual research projects.
- *Group reflections*: For the duration of the doctoral programme, the PhD scholars used self-facilitated group reflection to enhance their experiential learning. Group reflection took place immediately after each PPI panel meeting. Reflections were guided by the Gibb’s Reflective Cycle [36], developed to give structure to learning from experiences, and to reflect on learning and development. PhD scholars reflected on the process of embedding PPI throughout the doctoral programme as well as time committed to organising, planning, and conducting PPI panel meetings. The PhD scholars retained written records of the group reflections. Data from these written records were analysed using content analysis.

### Data analysis

Quantitative data from the PPI impact logs were summarised using descriptive statistics. The perceived impact of PPI on the individual research projects was summarised using qualitative data from the impact logs. Data from the activity logs was used to summarise the activities involved and to calculate the time commitment required for PPI activities as part of a PhD programme. For the analysis of qualitative data from the focus groups and interviews, a reflexive thematic analysis was adopted [37–40]. An inductive or ‘data-driven’ approach was used [38]. While an researcher external to the programme was engaged for the purposes of objectivity, the adoption of a reflexive approach for thematic analysis serves to highlight the active role that researchers play in producing findings. The subjectivity of the researcher is therefore acknowledged and viewed as integral to the process of data analysis. While the findings reflect the interpretations of the external researcher, data were ‘open-coded’ and semantic coding primarily used, allowing for the production of codes that were reflective of the content of the data and as communicated by study participants [38]. Data were interpreted using an experiential orientation to emphasise the perspectives of the PhD scholars, PhD supervisors and PPI contributors and prioritise their accounts of the programme [39]. The six phase analytical process proposed by Braun and Clarke was followed [41]. Other techniques utilised to increase the credibility of the findings included a longitudinal design with follow-up interviews/focus groups with study participants, triangulation of data, briefing the CDA-MM Steering Committee on the research process, testing the findings and interpretations with PhD scholars and with PPI panel members through member checking.

### Methods used for PPI in the evaluation

The PPI panel were consulted about plans for the evaluation. This was made possible by commencing the evaluation at 24 months, allowing time for the formation of the PPI panel. A clear strength of this approach is that it gave PPI contributors time to gain an understanding of PPI as a concept and how it worked in practice, before asking them to both participate in the evaluation as study participants and give their views on plans for the evaluation. With respect to evaluation planning, the PPI panel were consulted by PhD scholars about their preferred method for obtaining the perspectives of PPI panel members and questions to be asked. They decided to contribute their data using focus groups rather than through individual interviews. They identified the topics to be addressed by the evaluation and the questions used to guide their own focus groups. This discussion group was held to ensure that the PPI panel members were involved to some extent as contributors to decision-making on the evaluation design [42]. The findings from the analysis of their data was presented to the PPI panel members at a meeting [43] to ensure that the results resonated with them and to give them an opportunity to reflect on the results. This paper reports on the completed study according to the Guidance for Reporting Involvement of Patients and the Public 2 (GRIPP2) short form [44] [see Additional file 4].

## Results

This section reports on the findings of the evaluation study. It first reports on the findings from the process evaluation, organised by key themes. A key theme is that formal PPI training for PhD scholars is perceived to be critical. Key themes relating to experiential PPI training are concerned with how the PhD scholars formed and worked with a PPI advisory panel, the added value, time commitment, and important aspects of PPI meetings including atmosphere, culture, values and structure. These are followed by key themes linked to reflections on enhancing formal PPI training, strengths and limitations of using a PPI advisory panel and additional supports needed. Two final themes from the process evaluation concern PhD supervisors’ role and oversight of PPI, and support for PhD supervisors to maximise PhD scholar PPI learning. The results section then outlines findings from the impact evaluation including the perceived impacts on research projects, PhD scholar, PPI contributors and PhD supervisors. The evaluation findings were presented to the PPI panel who reported that they were accurate and resonated with their experiences and views.

### Formal PPI training for PhD scholars is perceived to be critical

Formal PPI training was regarded positively by PhD scholars and deemed a critical element for embedding PPI in the CDA-MM. Positive aspects were the interactive nature of the training, the practical, problem-solving approach adopted and the way in which the training was tailored to the needs of PhD scholars. The first two training workshops provided PhD scholars with the fundamentals for embedding PPI in research, which they described as beneficial and ‘grounding’.

“I think a large part of that was at the beginning we were all very task-oriented and training gave us a framework to hang our first meeting on and [PPI lead] talked to us about relationship management. It gave us a clear vision of what we should be working towards for the first meeting. But I also liked that [the PPI lead] targeted the training at the level that we were at. It was very well thought out.” [Focus group with PhD scholars]

Given their lack of experience, the PhD scholars initially felt apprehensive about conducting PPI and worried about doing it incorrectly or wasting the PPI panel members’ time. The workshops provided very practical information and were instrumental in helping allay concerns that the PhD scholars had, giving them the confidence to establish a PPI panel and organize meetings. An important aspect of the training for the PhD scholars was its focus on building and maintaining PPI relationships.

PhD scholars identified the workshop on facilitating PPI meetings online as being particularly important, especially as PPI meetings had to move online from March 2020 due to the Covid-19 pandemic. They described how the workshop provided practical information about how to keep the meetings interesting, interactive, and inclusive and equipped them with skills necessary for facilitating meetings and communicating effectively online.

PhD supervisors were in agreement with PhD scholars that formal PPI training is fundamental for embedding PPI in a structured PhD programme. They stressed its importance given that PPI is becoming more prominent and is increasingly a requirement of grant funding bodies.

“The training is actually fundamental to that process of actually knowing what PPI is and isn’t, especially given that grant awarding bodies are now insisting on PPI. It is very useful for our researchers to know what is and what isn’t PPI, so I think that is absolutely central.” [PhD supervisors, FG1]

Moreover, they stressed that formal PPI training was needed because of the complexity of PPI and the specific expertise required. Given its complexity and in recognition of PPI as a specific expertise, PhD scholars and PhD supervisors highlighted the importance of having formal PPI training and ongoing support from an experienced and capable PPI lead. The PhD scholars found the energy and passion of the PPI lead inspirational. Having a PPI lead involved in the CDA-MM was in their view critical to the CDA-MM’s success.

### Forming and working with a PPI advisory panel for experiential PPI training

In their first year of the CDA-MM, the PhD scholars worked together to establish a panel of eight people living with two or more ongoing chronic conditions to advise on their research projects. The PhD scholars set up the new PPI panel relatively quickly (within approximately 12 weeks), recruiting PPI contributors with two or more chronic conditions through charities, and professional and personal contacts. They wished to reflect the diversity among people with multimorbidity in the PPI panel, and succeeded in forming a panel of members who differed by age, gender, rural/urban location, were from different socio-economic backgrounds, and included a carer. However, identifying people who were eligible and reflecting diversity required an investment of time and effort and using many of their contacts. PhD scholars needed little support from PhD supervisors to create the PPI panel. Once established, the advisory panel was consulted with regularly throughout the research cycle from giving feedback on research plans and protocol development through to the sharing of research findings (Table 1). Accordingly, the advisory panel model used in CDA-MM can be best described as, and provides a good example of, ‘embedded consultation’^1^[18, 45].

**Table 1:** Range and impact of activities in which PPI panel members were involved

Category of activity	Number of PPI activities	Impacts	Challenges/comments
Research plan/protocol development (e.g., reviewing and commenting on importance of study, research questions, impact of Covid-19 pandemic, participant recruitment; involvement in protocol development)	2	On research plan: Confirmation that research is worthwhile and relevant Changes to language Contextual issues identified Greater clarity to protocol On PhD students: Boosted confidence in research project	Making changes to research plan may be limited due to stage of research and deadline for submission of ethics application
Intervention implementation (e.g., views on referral to link worker)	1	Changes to phrasing of information for potential trial participants with respect to reasons for referral for intervention	
Research ethics and data protection (e.g., providing advice relating to query on GDPR from HRCDC)	1	On ethics application: Likely to have contributed to a favourable response from HRCDC and REC	Not always easy to identify the actual impact of input from the PPI panel on some outcomes
Recruitment materials (e.g., reviewing and commenting on recruitment materials such as participant information sheet, consent forms, recruitment brochures, infographics; involvement in developing materials)	6	On recruitment materials Flyer and letter of invitation developed in addition to participant information sheet (PIS) and consent form Changes to and simplification of language in recruitment materials Changes to layout including location of GDPR related information in the PIS Clarifications made Likely to have contributed to good recruitment On ethics: Positive ethical approval outcome, likely in part due to the contribution of PPI panel to recruitment materials	There are limits to reducing PIS due to need to include detailed GDPR-related information, but where this information is placed in PIS is important Achieving consensus on whether to audio-record interviews The phrase ‘usual care’ seen as off-putting
Recruitment strategies and issues (e.g. incentivising people to take part; communicating study to potential participants; participation during Covid-19 pandemic; role of pharmacist in trial intervention; communication about pharmacist role in trial intervention)	5	On recruitment strategies Brought about use of pre-paid envelops for research participants Reassured researchers about planned recruitment strategies Addition of online recruitment strategies Led to greater choice in participation formats (online, face-to-face, telephone) Confirmation of services offered by the intervention Ways of informing patients about services offered in the intervention, likely to have had on impact on recruitment to trial	Limited flexibility in terms of uptake of ideas – dictated by ethics application/approval
Research instruments (e.g., reviewing and commenting on interview schedule; reviewing and commenting on questionnaires, using role play (whereby PPI contributors assumed the role of study participants) to assess questionnaire)	6	On research instruments Clarifications made Addition or reduction in number of questions Addition of prompts Ordering of questions	Constraints on broadening the research to include non-English speakers
Research findings (e.g., presenting of study findings for comment and feedback)	4	On research findings: Findings from qualitative study reflect experience of PPI panel Changes to phrasing Assisted in interpretation of findings from quantitative study Discussion of findings enhanced with new perspectives and insights On potential future interventions and research: Highlighted areas for future consideration, intervention development, research, and evaluation	Time constraints Time taken to explain findings
Dissemination of research findings (e.g., advice about sharing findings with lay audiences; reviewing and commenting on infographic)	2	Confirmation of importance of sharing research findings with lay audiences Mechanisms for dissemination identified Role of infographics in dissemination highlighted Importance of simple, short infographic with clear narrative and key findings that matter to public	
Total number of activities	27		

The PhD scholars selected discrete areas of their PhD projects in which they could readily involve the PPI panel. Table 1 summarises the different PPI activities in which the PPI panel were involved and the number of times they were involved in each activity type. In total nine PPI panel meetings were held between May 2019 and February 2022. Two additional meetings involved engaging some panel members in PPI activities using a one-to-one format. Including the one-to-one meetings, the PhD scholars initiated and involved advisory panel members in 27 discrete PPI activities. At each PPI panel meeting, there were between one and five PPI activities, with an average of 2.5 PPI activities per meeting. Each PhD scholar led between six and seven activities involving advisory panel members.

### Added value of experiential PPI training

The PhD scholars stressed that embedding experiential training in a structured PhD programme is essential for learning about PPI, and outlined the added value of running a PPI panel:

“To run a PPI panel … you can certainly get a grounding in it and you can get the basics and framework and the knowledge and motivation for it from training, but I think you don’t actually learn how to do it until you are doing it and have that responsibility. Experiential learning is what makes you become the expert in it. It is when you reflect on what happened, what worked well, what are the challenges, and you can hone and refine the process as you go through it. That would be the key thing for me.” [PhD Scholar 3]

Working with a PPI advisory panel was regarded by the PhD scholars as a successful model for a structured PhD programme, providing ample opportunities for experiential learning. It worked well for practical and institutional reasons; it was relatively straightforward to plan meetings, manage the budget and organise reimbursements for panel members.

From the beginning, the PhD scholars felt a very strong sense of responsibility for the advisory panel members. Working with the PPI panel required each of the PhD scholars to take on a leadership role. They had to maintain a balance between keeping in contact with panel members and at the same time respecting their time and private lives. A main concern for the PhD scholars was to manage relational issues well. According to PPI panel members, they succeeded in doing this by placing an emphasis on building and maintaining relationships.

“Worth pointing out that the four students put a lot of work into developing a good relationship with us from the outset.” [PPI panel member, FG1]

PhD scholars identified being respectful and having good listening and communication skills as important qualities for building and maintaining relationships with PPI contributors. They stressed that having a clinical background was not a prerequisite for demonstrating these qualities.

Collaborative working between PhD scholars was a key aspect of the advisory panel model. All four PhD scholars were proactively involved and shared responsibility equally. This helped to reduce the workload attached to planning, organizing, and conducting PPI meetings and promoted learning between the PhD scholars. It also introduced a peer support element for PPI, regarded by the PhD scholars as a ‘massive benefit’ and highly valued by them.

“I personally felt very supported particularly by the other students. The peer support is hugely important and would have been so difficult to do that on your own. I think a lot more mistakes would have been made. We were not perfect. We made lots of mistakes but being able to discuss and bounce ideas off each other and reflect afterwards on what worked well, what didn’t work well, what would we change for the next time. That was the most valuable support.” [PhD scholar 4]

They expected that without peer support PPI would have been much harder to do. The PhD scholars stressed the importance of group reflection after PPI panel meetings. They suggested that formally designating a PhD scholar to lead on the PPI panel, rotating over time, would be helpful.

### Time commitment for experiential PPI training

Time has been identified as the most significant cost of PPI from a researcher’s perspective [5], including by doctoral researchers [21]. Using sample activity logs, this evaluation sought to estimate the amount of time it takes to plan, organise, and conduct PPI panel meetings. Table 2 shows the activities undertaken by PhD scholars and the amount of time taken. The total amount of time spent for the two sample meetings was similar, 26.6 h for the first and 28 h for the second, an average of 27.3 h. The three most time-consuming activities were: the preparatory meeting and discussions among PhD scholars to design and plan the PPI panel meeting; preparation of materials for PPI activities at the PPI panel meeting; and time taken to attend and facilitate the meeting. Approximately one hour was spent contacting PPI panel members about the meeting. PhD scholars spent between 20 and 45 min each debriefing and reflecting on the PPI panel meeting. PhD scholars had strong administrative support for the administration of vouchers for PPI contributors, meaning that a relatively small amount of their time was spent on this task. Setting up IT for Zoom meetings was not considered time consuming relative to other PPI activities. However, outside of the meetings, one PhD scholar spent a lot of time supporting a PPI contributor who had little digital literacy.

**Table 2:** Acitivty logs for two sample PPI panel

Activity	Time commitment (mins) PhD Scholar A	Time commitment (mins) PhD Scholar B	Time commitment (mins) PhD Scholar C	Time commitment (mins) PhD Scholar D	Total time commitment (mins)
Activity log 1					
Preparatory meetings, discussions	90	130	140	120	480
Agenda preparation and review	10	35	10	10	65
Contacting PPI members	10	30	15	15	70
Preparation of materials, e.g., slides, infographics	210	120	60	–	390
Setting up and planning use of technology	–	–	–	10	10
Attendance at meeting	120	120	120	90	450
Debriefing and reflection	20	20	30	30	100
Organising vouchers	–	–	–	30	30
Total	460	455	375	305	1,595 min = 26.6 h

Activity log 2					
Preparatory meetings, discussions	55	65	60	120	300
Agenda preparation and review	–	–	–	–	–
Contacting PPI members	10	20	15	10	55
Preparation of materials (e.g., slides, infographics, leaflets, summary material) and circulation in advance of meeting	25	–	240	315	580
Setting up and planning use of technology	–	25	–	–	25
Attendance at meeting	120	120	120	120	480
Debriefing and reflection	45	30	45	45	165
Organising vouchers	–	–	–	30	30
Preparing minutes	–	–	45	–	45
Total	255	260	525	640	1,680 = 28 h

Note: Activity log 1 relates to one PPI meeting (conducted in first 12 months of study) Activity log 2 relates to one PPI meeting (conducted in final 12 months of study)

Assuming that planning, organising and conducting each of the PPI meetings takes 27.3 h, this equates to a total commitment of 245.7 h by PhD scholars over the course of the CDA-MM. Assuming that there are 7 h in a working day, this translates to a total of 35.1 days or almost 9 days (8.8 days) per PhD scholar. This equates to approximately one day of work per PhD scholar for each PPI panel meeting. Note that this calculation does not include the time involved for a single PhD scholar to plan, organise, and conduct a separate individual meeting with one or more PPI panel members. PhD scholars reported that such individual meetings take less time to plan and organise, estimated at approximately four hours per individual meeting. Nor does the calculation include the time spent by PhD scholars in the formation of the PPI panel, or the evaluation study.

As far as we are aware, estimates of the time it takes for other aspects of doctoral research projects are not available. There is a risk that producing estimates of the time it takes to embed PPI activities in doctoral research studies might reinforce a traditional view that PPI is not an integral part of the research project. However, producing such estimates serves to raise awareness of the time commitment for meaningful PPI so that it can be planned for effectively [5].

Time was a recurring theme in focus group interviews with PhD scholars and in their group reflections. PhD scholars identified time as the biggest challenge faced when embedding PPI in a structured PhD programme. In addition to the time it takes to do the practical planning and organization, PhD scholars pointed out that there was cognitive and emotional effort involved in PPI activities.

“People actually think about the time that the meeting takes, an hour every six weeks or so that’s not much but a lot more goes into that and a lot more cognitive and emotional energy goes into it. As [PhD scholar 1] was saying, you feel responsible for people so you are thinking about it even if you are not actually directly working on it all the time.” [Focus group with PhD scholars]

They stressed that while PPI is worthwhile because of the benefits, collaborating with other PhD students and building relationships with PPI panel members is time-consuming, something that may not be sufficiently accounted for in advance. They referred to 'tensions of time’ they experienced, with PPI activities competing with the many other time demanding aspects of a structured PhD programme such as attending structured elements of the PhD and writing papers. This suggests that adequate support for PhD scholars embedding PPI in research is essential for promoting their wellbeing [21]. Such support is necessary given that time pressures and time conflicts have the potential to cause undue stress among PhD scholars [46]. Support, including support for researchers, has been identified as a key value underpinning good PPI practice [47]. PhD scholars were always mindful of the burden of time on PPI panel members. However, reflecting on the time they had committed, the PPI panel members considered that it was very reasonable and well within acceptable limits:

“It is one meeting every two months, so it is not an excessive amount of time.” [PPI panel member, FG1]

One-to-one involvement was, however, experienced as more time demanding. For some PPI panel members, involvement granted a welcome opportunity to take time away from the routine of everyday life and a distraction from their health problems.

### Friendly, supportive, and inclusive atmosphere

For many of the PPI panel members, the CDA-MM was their first involvement in PPI in research and at the beginning they had been hesitant and reticent about getting involved. For these PPI contributors, PPI meetings were a new and strange experience:

“I didn’t know what I was going into, so naturally … it didn’t *feel* like a strange environment, it *was* strange, because I had never been in that environment before.” [PPI panel member FG1]

Feelings of anxiety, fear, uncertainty, and lack of confidence were common but quickly allayed by the warm welcome and reassurance received from PhD scholars and the explanations and clarity they provided. It can take time for panel members new to PPI to understand what PPI involves. Ongoing support from the PhD scholars in the form of checking in, positive feedback and reassurance continued to be important throughout the CDA-MM.

For PPI contributors, the culture of the meetings was an important aspect of the PPI meetings. The PPI panel described the atmosphere as relaxed, friendly, homely, and supportive. They found the environment to be always respectful, inclusive, and accommodating of diverse needs. They stressed the work of PhD students in building relationships from the start, and used the metaphor of family repeatedly, an indication of the strength of relationships. PPI contributors found that the students were genuinely interested in and open to their input. They reported that the PhD scholars listened sensitively to what they had to say and allowed them time and space to express their opinions, both of which were highly valued by PPI panel members.

“There is no sense at all that four students are just, as it were, going through the motions with this PPI group. They really are genuinely interested in hearing what we have to say and taking that on board and that reflects very well on them in that they are open to receiving input.” [PPI panel member FG1]

PPI panel members repeatedly stressed the importance of listening as a key element of the culture and felt that this was a skill that all of the PhD scholars had in abundance from the start and only improved over time. An important way in which PPI panel members gauged whether they had been listened to and their input taken on board was from the feedback that PhD scholars provided. They found it encouraging when they learned that PhD scholars had changed their projects in some way because of their contribution such as making participant information sheets more accessible.

“When they put something in front of us and we say ‘no, that is not how it works’ or ‘people won’t understand that’, you can’t help wondering at the time whether they will amend the language or their thesis but they usually come back to us and show us that they have and that reassures us that they are listening to us and taking our advice and if you began to see a pattern of them not doing it, I would be fading away thinking this is a waste of time, but to their credit they have.” [PPI panel member, FG2]

### Values and meeting structure

A key characteristic of the advisory panel model is that the research team maintains ownership and control over the research study whilst engaging in meaningful consultation with others. While PhD scholars in the CDA-MM were able to obtain the views of the advisory panel members, occasionally they could not commit to acting on their advice. This is an issue that has previously been identified as a disadvantage of consultation [48]. Initially, the PhD scholars found this to be challenging, exacerbated by some uncertainty and lack of clarity at the beginning of the programmme as to the scope of PPI activities and the extent to which research projects were open to change. Yet, it brought to the fore the issue of transparency and accountability, key values associated with PPI [12, 47, 49], and especially the importance of communicating clearly and effectively with advisory panel members about how and why decisions are made.

The PPI panel were highly satisfied with the advisory panel model and the level of engagement. They particularly liked breaking into small groups for discussions and working, as it gave them space and they felt more confident expressing their opinions to a smaller number of people.

“I thought the breakout rooms gave us more space. We all get more animated and contribute more fully and more enthusiastically.” [PPI panel member, FG2]

They preferred having something concrete to work on as opposed to working on abstract ideas or questions. Apart from becoming less formal and more relaxed, they reported that the panel meetings had remained much the same over time.

Online meetings, while used in doctoral studies before the Covid-19 pandemic [21] were more widely embraced during the pandemic to enable PPI to take place remotely [20, 24], including in the CDA-MM. PPI panel members highlighted several drawbacks of online meetings including technological know-how and issues, lack of opportunity for small talk, difficulty in knowing when to make a contribution and reluctance to share personal stories to illustrate a point. Despite these drawbacks, PPI panel members agreed that online meetings offered a good substitute when meeting in person was not possible due to the Covid-19 pandemic. Most importantly, it allowed the PPI panel meetings to continue. Another positive aspect highlighted was the potential to reach a wider group of people to involve, as has been previously highlighted [21]. However, the preference of all panel members was for in-person meetings.

“I would much prefer that we were all back in a room together rather than Zoom. It’s hard to convey or to interrupt a lot of the time when you are on Zoom. I think when we are back in a room together it will be much better.” [PPI contributors, FG1]

They believed that in-person meetings before public health restrictions were introduced helped cement relationships and make the transition to online meetings easier. Consistent with other studies [24], this study shows that it is possible for PhD scholars to build and maintain good working relationships with PPI contributors through regular online meetings, although this is supported by having pre-existing established relationships [50]. While there are both benefits and drawbacks to online meetings, the preference expressed by PPI contributors in the CDA-MM for in-person meetings suggests that in-person PPI meetings matter.

### Reflections on enhancing PPI training

The PhD scholars noted that their approach to PPI panel meetings developed over time. Their initial focus was on the practicalities of conducting meetings and the approach they took was largely didactic and centred on seeking confirmation or validation from the PPI panel. As they became more confident and experienced, they became more skilled in facilitating discussions at meetings. Nevertheless, group facilitation skills was a recurring theme in interviews with PhD scholars and in their group reflections. Despite formal and experiential PPI training, the PhD scholars often felt that their group facilitation skills were somewhat lacking and could be enhanced with further training and support. It was a skill they felt should be learned very early on in the process. One proposal for future structured PhD programmes was to incorporate observational learning into PPI training, whereby either PhD scholars would observe a PPI panel meeting in practice, or an experienced PPI lead or contributor would observe the PhD scholars as they facilitated a meeting and provide them with feedback on what could be improved and how to better engage with PPI panel members. They would also like to have learned more about different strategies for engaging with PPI contributors.

“I think the idea of having someone sit in and evaluate and just observe, maybe a patient representative who is very experienced at that work, and could tell us how we could involve people a little more deeply as well as researchers who have done it. But the public representative perspective would be really, really valuable. A person who has been very deeply engaged in research, what are we not doing.” [Focus group with PhD scholars]

The PhD scholars were always concerned about creating space for all PPI panel members to make a meaningful contribution. As their projects progressed, PhD scholars sought to do this by ensuring that PPI meetings were less didactic and more discursive and exploratory. This involved a lot of planning in advance of PPI meetings and using a variety of techniques such as discussion groups, breakout rooms, identifying particular as opposed to broad issues for discussion, providing material in advance and using role play. 

The PhD scholars frequently reflected on whether they could or should have achieved a greater level of engagement with the PPI panel or if they could have broadened the scope of PPI engagement, for example, to select or develop outcomes measures that matter to people with two or more chronic conditions. They also talked about ‘going deeper with PPI’ or ‘taking PPI to the next level’, suggesting that they were interested in moving towards what are arguably regarded as higher levels of PPI such as collaboration and coproduction or user led research. At the same time, the PhD scholars recognized that they were engaged in a process of experiential learning, learning how to do PPI in practice and about its benefits, and concluded that embedded consultation was likely to be most appropriate for PhD scholars inexperienced in PPI. Embedded consultation is ‘involvement where members of the public with relevant lived experience, are consulted with regularly throughout the research cycle from giving feedback on research ideas and proposals through to the dissemination of findings’ [45]. Key characteristics are the regularity and range of methods of engagement used, and while the research team retains ownership and control over the research study, engagement with PPI contributors is meaningful [45].

**Table:** 

Box 1: Recommendations for additional training to enhance formal PPI training for PhD scholars
• Provide PhD scholars with an opportunity to discuss the permissible level and scope of PPI in research projects and to reflect on issues of power and democracy in the approach to PPI adopted
• Include training on group facilitation skills early on in formal PPI training
• Consider incorporating observational learning into formal PPI training
• Provide training on different strategies for engaging PPI contributors
• Provide PhD scholars with training on how to manage tensions that may potentially arise

Reflecting on their experience, PhD scholars would have liked to have had a greater opportunity to explore the level and scope of PPI in more detail with PhD supervisors at the beginning of the programme. PhD scholars also reflected on what they could potentially do when they engage with PPI in the future. In this way, experiential learning through consultation with the advisory panel was preparing them for embedding PPI in future research. It also brought to their attention broader issues of power within PPI and democratic ideals of PPI. Recommendations for enhancing formal PPI training in a structured PhD programme are provided in Box 1.

### Strengths and limitations of using an advisory PPI panel in a structured PhD programme

In focus groups, interviews and group reflections, the PhD scholars deliberated on the advantages and disadvantages of adopting an advisory panel approach for embedding experiential PPI training in a structured PhD programme. A strength of the advisory panel model is that it enabled PhD scholars to understand the experiences, views and perspectives of a number of people as opposed to relying on one person [45]. However, the depths to which PhD scholars could explore an issue at an advisory panel meeting was on occasion limited due the number of people in the group and the time constraints of the meetings. There is also a limit to the number of PPI research activities that can be accomplished at an advisory panel meeting. Where one advisory panel is concurrently working with four PhD scholars, this can on occasion give rise to scheduling challenges, as PhD scholars may be vying with each other for time to carry out PPI activities. While this issue was not experienced in the CDA-MM, it could potentially be a challenge in a structured PhD programme. Perhaps more importantly, it highlights the significance of careful planning to synchronize the PPI activities from each of the four individual research projects to the advisory panel schedule and the important role that strong collaboration and coordination between PhD scholars plays.

The PPI panel believed that an advisory panel offers a suitable model for a structured PhD programme. They liked and found it interesting to be contributing to four projects on the theme of multimorbidity. However, it took some time for at least some panel members to understand the individual projects and link them with PhD scholars. While PhD scholars had at the beginning presented the PPI panel with an overview of their projects, some panel members had missed this meeting and struggled to get an overall sense of the projects and how points discussed at meetings slotted into the ‘bigger picture’ of what each PhD project was trying to achieve. They felt it would be helpful to have an overview repeated at intervals and would also have liked an overview of the background to CDA-MM such as how it had come about and how the projects had been decided and PhD scholars recruited.

Over time the advisory panel evolved slightly with some PhD scholars holding a small number of individual meetings with one or more members of the PPI panel. PhD scholars felt introducing more flexibility would be beneficial for overcoming certain limitations associated with the advisory panel model. They suggested that, if resources allowed, perhaps a hybrid model may be useful for future structured PhD programmes. Such a model would comprise an advisory panel from which four PPI contributors would be drawn voluntarily to each work closely with one PhD scholar and get to know and understand the project in depth, as has been adopted by CDA-iPASTAR, a PhD programme for stroke care [19]. In their first year (2021), PhD scholars on CDA-iPASTAR met with and received advice from CDA-MM PhD scholars. PhD scholars acknowledge that there may be funding and time implications associated with a hybrid model [19]. They had some concerns that a hybrid model could potentially be more challenging to manage, and suggested that it would be helpful for PhD scholars to receive advice on how to manage such tensions should they arise.

PPI panel members had mixed views about making changes to the model. Some didn’t see any reason to change it in any way: ‘there was no need to fix what wasn’t broken’, whereas others believed that it might be easier to work on one individual project rather than four and there was some interest in the hybrid model mentioned above.

PhD supervisors acknowledged the benefits of group working and peer support, and challenges of managing time. They suggested that there may be some benefit gained from introducing a contained level of flexibility to the advisory panel model. However, they warned against PPI becoming overly regimented or standardised, and stressed the importance of avoiding a cookbook approach to PPI in research.

“I always think that PPI is case by case, it is not standard, like I’m always concerned about it being a box ticking exercise like we have to do it and every project has to have it and they all have to have it in the same way.” [PhD supervisors, FG 2]

Of most concern to PhD supervisors, irrespective of the model adopted, was ensuring that meaningful PPI was undertaken that gave PhD scholars opportunities to take ownership of the research and to begin to make judgements, take decisions and solve problems on a range of issues from early in the research process and to learn transferable skills.

### Support for PhD scholars during experiential PPI training

PhD scholars identified the ongoing support from the PPI lead during their experiential PPI training as an important and invaluable resource, especially in a context where they knew of no other PhD scholars embedding PPI in their research projects. Over time, the PhD scholars came to rely more and more on peer support and less on support from the PPI lead. Reflecting on this in interviews, they felt that perhaps they had come to rely too much on peer support and recommended that a greater level of structured support and mentoring by a PPI lead throughout be a feature of future experiential PPI training..

With respect to supervision, the PhD scholars noted that early in the programme, they had tended to compartmentalize their PPI activities leading to a disconnection between these and other PhD research activities. They were aware that PhD supervisors were not all experienced in PPI to the level that PhD scholars were implementing it, e.g., forming a PPI panel, facilitating regular meetings. This meant that at first in PhD supervisory sessions, PhD scholars would only discuss PPI activities in passing with their supervisors. As their confidence grew both with respect to PPI and as researchers, and as they started to see the benefits of PPI, they began to take the initiative to report back in more detail on PPI activities and how these were changing their project, leading to greater discussion around PPI in supervisory sessions.

“I might tell them if we had a PPI meeting since the last supervisory meeting but it was never something that there was much discussion on or it wasn’t like they were ever pushing me to say ‘well have you ever thought about how you might engage the PPI group in this?’” [Focus group with PhD scholars]

“… now I find that it is almost a natural part of the PhD supervision that if something comes up perhaps it might be worth bringing to the PPI panel and we can discuss at the next meeting how you get on with them. I think it has evolved over time and has become a natural part of the conversation.” [Focus group with PhD scholars]

The PhD scholars welcomed that PPI was a requirement of the HRB grant. Without the funding, it would have been difficult for the PhD scholars to establish and run the PPI panel. The availability of funding meant that the PhD scholars did not encounter the financial limitations that have been reported by doctoral researchers [21]. However, funding on its own was not sufficient for the PhD scholars to undertake high quality PPI.

The structure of the CDA-MM, designed with a dedicated and knowledgeable PPI lead, was central in enabling the PhD scholars to learn about and put PPI into practice.

### PhD supervisors’ roles and oversight of PPI

In previous studies doctoral students have emphasised the important role that PhD supervisors play in encouraging and facilitating PhD scholars to embed PPI in their doctoral studies [18, 22, 25]. As far as we are aware, this is the first study evaluating PPI in a doctoral programme that includes the perspective of PhD supervisors. PhD supervisors in the CDA-MM had varying experiences of applying PPI in practice and over the past five to ten years had increasingly been exposed to PPI as it had become more prominent. PhD supervisors shared a commitment to avoiding tokenism and promoting and encouraging meaningful PPI in research. They identified three clear roles for PhD supervisors in supporting PhD scholars to embed PPI in their research projects and to foster meaningful PPI. First is to be a positive role model, which involves being supportive of PhD scholars embedding PPI in their studies.

“ ... I think role modelling is really an important part of being a PhD supervisor and I have heard senior academics dismissing PPI as it is just a tick-box, it is tokenistic, and they are people who have never actually done it. So, I think the fact that a supervisor is even positive about it is a really important experience for a PhD student to have; they are seeing it as something as valuable to the people I am trying to be like.” [PhD supervisor, FG 1]

Second is to utilise PPI as a means of encouraging PhD scholars to become critical thinkers, thinking critically about their approach to and use of PPI in research.

“Even though we may be very supportive of it, I think it [PPI] is a way to communicate that [critical thinking] and challenge and interrogate PhD students, as well as in terms of how they are using the approach. Because very often you get … a lot of people, a lot of us … can be half-hearted in a way of PPI but equally it can go the other way and can become an advocate, potentially see PPI as being almost in terms of a hierarchy of engagement, of being at the top end, so I think you have to interrogate really critically, that will ensure that people are not doing it to fulfil some grant awarding body’s requirement, but they actually see well whether PPI they are doing is good or bad …" [PhD supervisor, FG 1]

Third is to train students in good judgement. An example of where this can happen is when dilemmas arise because of PPI panel input such as when a consensus is not reached between PPI contributors and PhD scholars on an issue. It is through these roles, which are typical of approaches used by effective PhD supervisors [51], that PhD supervisors can encourage PhD scholars to embed PPI in their doctoral research in a meaningful way.

There may also be a role for PhD supervisors in relation to the quality of PPI and ensuring that the process is facilitating meaningful engagement. In the context of the CDA-MM, PhD supervisors played a lesser role in this regard since the Programme lead and PPI lead took responsibility for ‘light touch’ oversight of the PPI process. In addition, the PPI process was being appraised as part of the evaluation study. Although PhD supervisors were somewhat removed from the work of the PPI panel, and despite slight concerns initially that the PhD scholars might approach PPI as a ‘tick-box’ exercise, they had a sense from interactions with the PhD scholars during supervisory sessions that PPI in the CDA-MM was being conducted authentically and was of high quality.

"I was wondering if it [PPI] would be one of those things that they [PhD scholars] just felt that they had to do but it’s really not. They have really taken hold of that group and we had a meeting yesterday with one of the PhD students who in the meeting, unprompted by us, said ‘that is something that I would like to bring to the PPI group, but I don’t want to bring it to them if it is not going to be something that they can contribute to.’ To be conscious of not wanting to waste their time and conscious of wanting to keep them involved in the project with such buy-in and really reflecting on whether this was the appropriate point for PPI or not." [PhD supervisors, FG 1].

There was also an awareness among PhD supervisors of the potential for PPI to cause harm to PPI contributors and PhD scholars. For these reasons, having PPI oversight in place at a programmatic level that is not overly bureaucratic, and providing feedback and reassurance to PhD supervisors in relation to the PPI process in a structured PhD programme are important.

### Supporting PhD supervisors to maximise PhD scholar PPI learning

At the request of some PhD supervisors, the PPI lead facilitated a PPI workshop arranged mid-way through the programme for PhD supervisors. This workshop was designed to be informal and take account of the varying levels of experience of PPI among PhD supervisors. It was particularly helpful for PhD supervisors who were less experienced in and less confident about PPI. As well as receiving formal instruction, the workshop provided a welcome opportunity to reflect on how PPI was being embedded into the CDA-MM, on how supervisor confidence in using PPI had grown, and for discussion on broader issues related to PPI. PhD supervisors are mostly learning about PPI not so much through formal training but through attendance at PPI seminars and conferences, incorporating PPI into grant applications, doing PPI and from ‘more experienced others,’ which for PhD supervisors less experienced in PPI may include PhD scholars and post-doctoral researchers. Given that PPI is a specific expertise and is a complex and evolving field, they raised questions about the appropriateness of training PhD supervisors in PPI vis-à-vis the importance of having a knowledgeable PPI lead involved in a structured PhD programme who is available to offer guidance as and when needed. The latter was regarded as especially important.

“… even with [ten years of] experience I would still refer to people that I would respect as having a lot more knowledge of [PPI] and that interface and particularly when I should use PPI contribution, when it is optimally best to use that contribution, which will of course differ across people, because it may be that you want it more in dissemination rather than methodology or vice versa, and I would say that view hasn’t changed very much.” [PhD supervisors, FG2]

It was suggested that the aim should not be about training PhD supervisors in PPI per se but about supporting PhD supervisors with a view to maximising student learning. It was believed that there would be merit in having a working group of PhD supervisors within a structured PhD programme seeking to embed PPI.

### Impacts on projects and key stakeholders

#### Impacts on research projects

At the beginning of the CDA-MM, the PhD scholars were unsure about how the advisory panel could potentially impact their PhD projects. Through their experience of embedding PPI in their research project, they all agreed that it is highly beneficial and can have a positive impact on research. Table 1 summarises the impacts the PPI activities had, as described by PhD scholars in impact logs, on the research projects. Each of the activities had at least one positive impact on the research. The activities impacted on many different aspects of individual PhD projects including directly impacting on research plans and protocols, intervention implementation, ethics applications, recruitment materials, recruitment strategies, research instruments, research findings and dissemination. For example, with respect to recruitment materials, a decision was taken to devise a letter of invitation and flyer in addition to participant information sheet and consent form, language in recruitment materials was changed to improve clarity and the layout altered following input from PPI panel members (see Table 1 for details of other impacts).

There were 22 records of the extent of impact of PPI activities, as self-rated by the PhD scholars. In almost two-thirds (14/22) of cases, the PhD students rated the level of impact as small and in close to one-third (7/22) as moderate. In one instance (5%), the PPI activity, which was related to recruitment materials, was rated as having a large impact on the research project, as the input of the PPI panel led to the development of an additional Study Within A Trial (SWAT). The overall low or moderate level of impact was in line with expectations of PhD scholars and PhD supervisors.

The impact log, while sometimes regarded by PhD Scholars as a tick-box exercise, proved to be an important record of PPI activities and their impact. It was useful as an *aide memoire* when PhD scholars were writing up the research for publication. It allowed PhD scholars to compare the impact of PPI on different research projects. No negative impacts were identified. However, the entries reveal several practical challenges that the PhD scholars encountered when embedding PPI in the projects such as the limits to which they can take on board the suggestions of PPI panel members or the extent to which it is possible to know for certain what impact the PPI panel has had on different aspects of the research. For example, PPI had likely contributed to positive outcomes such as a favourable response from Health Research Consent Declaration Committee (HRCDC) and from research ethics committees or had contributed to successful recruitment of research participants, but the extent to which it impacted these outcomes is hard to gauge.

According to PPI panel members, the biggest impact they had on PhD research projects related to language:

“There were times that I thought what they were putting in front of us was way out, you know, it was very scientific. You’d put that to them and they would certainly take that on board. The language was very scientific, and you would have to say to them ‘This is too long, it is too scientific and the public won’t understand that’.” [PPI panel members, FG 1]

#### Perceived impacts on PhD students as researchers

Prior to joining the CDA-MM, most but not all of the PhD scholars had been exposed to the idea of PPI either through clinical training where patients were involved as educators or through their work in an organization seeking to set up PPI structures. None of the PhD scholars had received any previous formal training or had any prior experience of conducting PPI in research. Despite their lack of training and experience, the PhD scholars were interested in and enthusiastic about PPI and, although apprehensive, were motivated to embed PPI in their PhD projects.

Embedding formal and experiential PPI training in the CDA-MM impacted on the PhD scholars as researchers in several ways. PhD scholars gained knowledge of PPI from both formal and experiential PPI training. Through experiential training they gained confidence in practicing PPI, as has been previously reported [22], and developed transferable skills including organisational and project management skills.

Experiential PPI training demonstrated to them the value of the patient perspective informing the research throughout. It put their PhD research into perspective, encouraging them to think more deeply about what they were trying to achieve and the impact that it would have on people with multimorbidity, and justify why they were doing the research. It kept them ‘grounded’ in the ‘real world’.

"You can get so embedded in it [research] and to have these experts to bring you back to why you are doing it, why it is important, what the focus of your research actually is, it is not about explaining and statistics and x, y and z, which is part of it, but the focus of your research is the patient, what impact is this going to have on their lives and why am I actually doing this at the end of the day. So having that grounding from them has been lovely. It’s been that connection with the real world through their lived experience of what we are researching." [Interview with PhD scholar 4]

The PhD scholars developed communication skills and group facilitation skills. Learning how to better communicate their research to the PPI panel helped with communication to wider audiences. They have developed relationship building skills; a major impact identified was the way in which PhD scholars approached PPI, with the relationship between them and the PPI panel changing from being didactic to one based more on partnership.

"We are partners with our PPI panel. We are very respectful and love getting their opinions particularly because it changes our research and our communication and improves it. I think that transition in the relationship has been one of the biggest impacts on me as a researcher." [Focus group with PhD scholars]

Through their work with the PPI panel, they had gained new perspectives on multimorbidity, for example, becoming aware for the first time of the multiple burdens experienced by people with two or more chronic conditions. An unexpected impact was that the PPI panel helped PhD scholars make linkages between different PhD projects, for example highlighting how the findings from one project related to the aims of another. PhD scholars had also become more skilled in both critically reflecting on and appraising PPI in research. PhD scholars could not imagine doing research in the future without PPI. While they would like to start PPI early in the research process, involving the public or patients in identifying research topics or questions, they were aware of the constraints of doing so.

The impacts outlined above supports findings from other research showing the benefits of incorporating PPI for doctoral students [18, 20–22, 24]. It has shown that the added value of experiential PPI training is wide-ranging. As well as enhancing PhD scholars’ understanding of and skills to conduct meaningful PPI, it contributed to the development of their leadership, project management and other important transferable skills.

#### Impacts on PPI panel members

In the focus groups, PPI panel members were asked about their perceived impact on research projects and on the PhD scholars as researchers. However, PPI panel members also spoke about the impacts that involvement had on them. They spoke about how their self-confidence had grown, and for some, increased self-confidence had an impact beyond the PPI panel meetings. Making a contribution had a positive impact on sense of wellbeing for some and was described as ‘empowering’:

"When you are dealing with an underlying health issue, you get labelled by just that title, so it is nice to feel that I am contributing in a positive way. I like the idea of being able to do that." [PPI panel members, FG1].

Knowing their contribution was having an impact on the PhD scholars’ research projects gave the PPI panel members a sense of achievement. They not only felt that they have achieved something as individuals, but also collectively as a group. They had a sense of being part of something important. While the PPI panel members believed that the PhD scholars had learned a great deal from their contributions, they reported that learning had been a ‘two-way process.’ They had learned from listening to each other, practical skills such as using Zoom and conducting online meetings, and, more broadly, being involved in the PPI panel had provided them with ‘a window into research’ that informs service development that is generally invisible to patients:

"We also learned, which we hadn’t thought about before, is that a lot of thought goes into services. For example, at GP level people are thinking all the time ‘how can we make this better?’ and real research is going into it. That’s good to know, because you take a lot for granted that things just appear or sometimes you are not satisfied with what is going on, but it is reassuring to know that real serious research is going on to try to make services better and the research itself is very interesting." [ PPI panel members, FG1]

Panel members perceived that involvement had also helped them to stay or become more cognitively active. All those participating in a second focus group or interview expressed interest, attributed to their positive experience, in being involved in PPI work in the future. However, some would like a break between projects, and some would like projects to be better matched to their own interests.

#### Perceived impacts on PhD supervisors

There was some evidence from the focus groups that embedding formal and experiential PPI training in the CDA-MM positively impacted on PhD supervisors but in varied ways. These included becoming more confident with PPI and supervising PhD scholars embedding it, developing greater awareness of the possibility to integrate PPI in different aspects of research and throughout the research process as well as earlier in the research process, helping to consolidate understandings and views on PPI, as well as changes to their practice.

“I have more of an eye on PPI and maybe I’m thinking about introducing it earlier in the research process than I did in the past. I think some of the things we did in the past that would be classified as PPI was probably much later in the research process. Recently we have definitely consulted PPI prior to writing grants. That would be something that is coming from [name of PhD scholar] and the CDA-MM, which makes you think about PPI very early in the research process.” [PhD supervisor, follow-up interview]

Through the CDA-MM, PhD supervisors have gained a greater appreciation of the nuances of PPI and how much there is to learn and consider. It also brought to the fore the need for PhD supervisors to view PPI through a critical lens. A broad range of questions and issues concerning PPI in research generally, but not specially related to embedding PPI in a structured PhD programme, were raised. These included issues of representation, diversity, and inclusion of marginalised groups of people, professionalisation of PPI contributors, the involvement of stakeholders other than patients, the optimum length of time for a PPI panel to remain in place, and questions regarding understandings of PPI effectiveness and impact and the complex ways in which health is produced. The impacts for PhD supervisors were unanticipated at the beginning of the evaluation.

## Discussion

The CDA-MM provides a novel example of a doctoral training programme that embedded formal and experiential PPI training in its structured education. The findings from this evaluation of the programme support the argument that, alongside other research skills that are required, it is beneficial to have PPI training included as a fundamental component in post-graduate education, as well as education for early career researchers [17]. A number of recommendations can be drawn from this evaluation for embedding formal and experiential PPI in a structured PhD programme. These are discussed below and summarised in Box 2. Also outlined below are key messages for PhD scholars seeking to embed PPI in doctoral studies (see Box 3),

### Recommendations for embedding formal and experiential PPI in a structured PhD programme

A strongly held view was that the inclusion of a PPI lead positively facilitated PPI to be successfully embedded in the CDA-MM. This is consistent with emerging findings from other studies [52]. A practice implication is when designing a governance structure for a structured PhD programme, it is advisable that a dedicated PPI lead with the appropriate skills and experience is involved as a member of governance structures, e.g. the Steering Committee. The PPI lead needs to be available throughout the lifetime of the programme and be able to: provide advice and guidance to the Steering Committee, PhD scholars and their supervisors; coordinate PPI training; and assist in the development and implementation of plans by PhD scholars to involve PPI contributors.

A key concern of all evaluation participants was to ensure that tokenistic and tick-box approaches to PPI were avoided in doctoral research. The qualitative feedback from study participants indicated that PPI in the CDA-MM succeeded in avoiding tokenism as a result of the time taken by PhD scholars to build good relationships with PPI contributors, foster mutual respect, and give feedback routinely to PPI contributors. The provision of formal PPI training was a key enabler of this as was the presence of a PPI lead to offer advice and guidance for experiential PPI training, although a key message is that the latter could be more structured. Other enablers were that the governance structure had a clear, well-defined vision supportive of the implementation of meaningful PPI, including with respect to the type of patients to be involved and how they were to be involved. In addition, the whole team were supportive of PPI. These are important as embedding PPI in research is contingent on having a clear purpose and role for PPI, and requires the whole team to be supportive of PPI [53]. Involvement of PhD scholars in setting the vision would help to provide further clarification around its implementation. Also important for fostering meaningful PPI is that PhD supervisors apply the approaches typically used in effective PhD supervision [51] when supervising PhD scholars embedding PPI in their doctoral research, with opportunities to have a facilitated discussion and collectively reflect on embedding PPI in a structured PhD programme and broader issues related to PPI. This could potentially be offered through a PPI workshop for PhD supervisors early in the programme and/or the formation of a PhD supervisors’ group (Box 1).

Adequate funding enabled the PhD scholars in the CDA-MM to embed PPI in their doctoral students. This is consistent with findings from other studies relating to PPI in health research [54, 55] and PPI in doctoral research [18, 20–22]. This highlights the important function of governance structures for ensuring that adequate funding is secured and allocated for PPI for the lifetime of a structured PhD programme. In addition, time allocated for PhD scholars to embed PPI needs to be realistic.

**Table:** 

Box 2: Recommendations for embedding PPI in a structured PhD programme
• Have an experienced PPI lead involved in the structured PhD programme
• Incorporate formal PPI training for PhD scholars as a fundamental component alongside training on other research skills
• Make dedicated funding available for PhD scholars to embed PPI experiential learning in their doctoral studies
• Consider the range of additional supports required by PhD scholars embedding PPI in their doctoral studies (including infrastructural supports, ongoing structured support from a PPI lead, PhD supervisors supportive of meaningful PPI)
• Consider introducing a PhD scholar peer support element
• Avoid careless repetition of approaches to embedding PPI in a structured PhD programme
• Consider the level of oversight of PPI required for a structured PhD programme on a case-by-case basis, and review over the course of the programme
• Consider offering a PPI workshop to PhD supervisors and/or forming a PhD supervisors’ PPI group. This could be organised by PPI leads or teams (e.g. PPI Ignite Network) and offered to a subset of supervisors on structured PhD programmes
• Consider PPI at grant writing stage
• Evaluate the process and impact of embedding PPI in a structured PhD programme to capture lessons learned. There is value in using longitudinal mixed methods to achieve this

Accountability/transparency is a key value associated with PPI [12] and doctoral researchers has shown how these principles can be put into practice [20, 22]. While PhD scholars will have responsibility for accountability and transparency throughout their studies, this evaluation draws attention to measures of accountability at the doctoral programme level, which has received little attention to date in the academic literature. It is advised that an oversight committee for PPI involving a PPI lead is formed. In the CDA-MM, only ‘light touch’ oversight of the PPI panel and experiential PPI training by the programme’s Oversight Committee was required. This might not always be the case, and more oversight may be needed if PhD scholars are inexperienced or where problems arise within or between PhD scholars and PPI contributors. Therefore, it is recommended that consideration is given to the level of oversight for PPI in a structured PhD programme at the beginning and is kept under review (Box 1).

The newly established advisory panel for the CDA-MM, was perceived to have worked well by those involved. The sharing of one PPI advisory panel by four PhD scholars in the CDA-MM offered real benefit in the form of peer support. However, this study warns against PPI becoming standardized in structured PhD programmes (Box 1), and approaches should be tailored to research questions and context, in keeping with Dawson et al. [22]. This may be achieved by building time into formal PPI training for PhD scholars to discuss the level and scope of PPI in research studies and issues of power and democracy with PhD supervisors early on in the programme. Flexibility can be introduced into this approach, and if introduced, PPI contributors need to be given the option of choosing the extent and level of their involvement.

Perhaps one of the greatest PPI impacts on the research happened during the grant writing stage before PhD scholars had been recruited to the programme. At this stage, PPI contributors provided direction as the PhD projects were being developed, which helped to shape research questions and led to the inclusion of a PhD on the topic of health economics with a patient perspective. It highlights how valuable it is to include PPI contributors when developing research projects and questions for a structured PhD programme.

### Key messages for PhD scholars seeking to embed PPI in doctoral studies

Key messages for PhD scholars seeking to embed PPI in doctoral studies are outlined in Box 3. These supplement pragmatic recommendations previously offered to PhD scholars [21]. For example, supporting findings from other studies on PPI in doctoral research [20, 22, 23], a key message is that the process of involvement, including building and maintaining trust and relationships, and the values and principles guiding involvement are of utmost importance when embedding PPI in doctoral studies, as they are in research generally. The CDA-MM offers examples of ways in which the six values and principles in the PPI Ignite Network Values and Principles Framework [49], can be implemented in practice. For example, the value of equity and inclusion is illustrated by the PhD scholars reaching out to and involving PPI contributors from a range of backgrounds.

**Table:** 

Box 3: Key messages for PhD scholars embedding PPI in doctoral studies
• Seek advice from a PPI expert, bearing in mind that an array of strategic and pragmatic decisions will need to be taken both before any PPI activity commences and as PPI is being practiced
• The process of involving the public and patients in research is important and includes ensuring that PPI, in practice, is underpinned by values and principles of PPI in research. Be guided by a Values and Principles Framework such as that devised by the PPI Ignite Network [49]
• Be prepared to initiate discussions with PhD supervisors about PPI activities
• Critically reflect on decisions taken and the process of involvement on a continuous basis
• Consider PPI as a social practice of dialogue and learning between PhD scholars, PPI contributors, PhD supervisors and PPI leads
• Conducting meaningful PPI in doctoral studies is time-consuming, and it is important to have adequate supports available to you
• Consider introducing a PhD scholar peer support element
• Consider the pros and cons of online vis-à-vis in-person PPI meeting, in consultation with PPI contributors
• Keep a log documenting the impact of PPI activities on the related research and PhD scholars practice
• Using mixed methods to evaluate the process and impact of embedding PPI can help to capture lessons learned

During the planning of this evaluation, there was dialogue with the PPI panel, reflecting the view in the CDA-MM programme of PPI as a social practice of dialogue between researchers and the public [56]. A positive effect of using this approach was that the PPI panel members could collectively reflect on their experience of being involved in a PPI advisory panel and valued hearing each other’s views. In consideration of the finite sample and dual roles of all study participants and in order to maintain a clear distinction between the research participant role and stakeholder role [1], it was decided not to engage in inter-stakeholder dialogue during data collection and analysis. The aim, in doing so, was to distinguish this research piece from usual PPI activities, where PhD scholars regularly engaged in dialogue and learning through reflection amongst stakeholders, including during the development of this evaluation study, and following regular PPI meetings.

### Strengths and limitations

This evaluation study has a number of strengths. The study makes a unique contribution to the literature in its evaluation of the process and impact of embedding PPI in a structured PhD programme. The process and impact of embedding PPI is evaluated from multiple perspectives using quantitative and qualitative research, providing a comparison of the different perspectives of PhD scholars, PPI contributors and PhD supervisors, and illuminating issues that might otherwise have gone unnoticed. Designing a mixed methods study utilising both qualitative and quantitative methods allowed for triangulation, widening the scope of conclusions that could be derived from the findings. Reflecting the mixed methods approach utilized, the results have been presented in an integrated manner, making it possible to clarify and elaborate on the quantitative results using qualitative findings and to generate additional information. The inclusion of in-depth qualitative methods was important for capturing rich and valuable subjective learning. A strength of this evaluation study is that it was led by an independent researcher (MP) who was otherwise not associated with the CDA-MM and not involved in CDA-MM governance, for reasons related to objectivity, credibility, and ethical considerations.

A limitation of the study is that the evaluation was completed before the formal end of the CDA-MM programme. While this ensured time for data analysis, engagement with the PPI panel about results and report and paper writing, it was not possible to collect data on what happened to the advisory PPI panel at the end of CDA-MM and what issues, if any, arose. Normalisation Process Theory or similar could be used for data analysis, but for this project, the qualitative data analysis was undertaken by an independent researcher who adopted an inductive or ‘data-driven’ approach. Another limitation is that the PPI lead’s experience and views were not explored in an interview for this evaluation.

## Conclusions

Beneficiaries of this work include health research funders, PPI leads and networks in higher educational institutes, governance structures of structured PhD programmes, PhD supervisors, PhD scholars and PPI contributors. Increasingly, PPI is mandated in funding applications for structured PhD programmes. Building capacity among early career researchers is crucial, but if not carefully considered, there is a danger that PPI in doctoral studies can become tokenistic. The Collaborative Doctoral Award in Multimorbidity (CDA-MM) programme provides a novel example of a doctoral training programme that has embedded formal and experiential PPI training in its structured programme. It offers a practical example of optimal approaches that can be taken by programme governance structures when seeking to ensure PPI activities are embedded in a meaningful way. Formal PPI training is a critical component and based on lessons learned, this study has suggested ways in which formal PPI training in a structured PhD programme can be enhanced to support implementation of PPI by early career researchers. The study has identified a number of key messages to supplement existing pragmatic guidance for PhD scholars engaged in experiential PPI learning. These are all relevant and can with careful consideration be translated to international contexts.

### Supplementary Information

**Additional file 1**. Good Reporting of a Mixed Methods Study (GRAMMS) guidance framework

**Additional file 2**. Activity Log Template

**Additional file 3.** Impact Log Template

**Additional file 4.** Guidance for Reporting Involvement of Patients and the Public 2 (GRIPP2) short form

## Data Availability

Not applicable.

## Abbreviations

CDA-iPASTAR: Collaborative Doctoral Award in Improving Pathways for Acute STroke And Rehabilitation Programme
CDA-MM: Collaborative Doctoral Award in MultiMorbidity Programme
FG: Focus Group
GRAMMS: Good Reporting of a Mixed Methods Study
GRIPP2: Guidance for Reporting Involvement of Patients and the Public 2
HRB: Health Research Board
HRCDC: Health Research Consent Declaration Committee
IRC: Irish Research Council
PPI: Patient and public involvement
SPHeRE: Structured Population Health and Health Services Research and Education Programme
SWAT: Study Within A Trial

## Footnotes

1 Embedded consultation is one of five operational definitions identified by Hughes and Duffy (2018) that can be used to guide, develop, and evaluate public and patient involvement in research. The five operational definitions of PPI in research are: undefined involvement; targeted consultation; embedded consultation; collaboration and co-production; and user-led research.
