# Supplementary material for: Embedding formal and experiential public and patient involvement training in a structured PhD programme: process and impact evaluation
Source: Res Involv Engagem. 2023 Nov 24;9:105. doi: 10.1186/s40900-023-00516-4 (PMC10668398; doi:10.1186/s40900-023-00516-4)
Supplement: Supplementary file 1 — Additional file 1. Good Reporting of a Mixed Methods Study (GRAMMS) guidance framework [file 40900_2023_516_MOESM1_ESM.docx]

**Additional File 4**

**Good Reporting of a Mixed Methods Study (GRAMMS) guidance framework**

| **Guideline** | **Section: page** |
| --- | --- |
| Describe the justification for using a mixed methods approach to the research question | Strengths and limitations: pg. 20  Designing a mixed methods study utilising both qualitative and quantitative methods allowed for triangulation, helping to increase the credibility and validity of the findings. |
| Describe the design in terms of the purpose, priority and sequence of methods | Methods; Study design: p. 4  A convergent parallel mixed method design was used, whereby the qualitative from different sources and quantitative data were analysed separately and then the results compared to see if the one supports or does not support the other |
| Describe each method in terms of sampling, data collection and analysis | Methods pp. 4-5 |
| Describe where integration has occurred, how it has occurred and who has participated in it | Methods; Study design: p. 4  The qualitative and quantitative data were analysed separately and then the results compared to see if the one supports or does not support the other. The integration of the data was undertaken by independent researcher (MP) with other authors providing feedback on results reported.  Strengths and limitations: pg. 20  Reflecting the mixed methods approach utilized, the results have been presented in an integrated manner. |
| Describe any limitation of one method associated with the present of the other method | Strengths and limitations: pg. 20  A limitation of conducting online focus groups was that a very small number of study participants were either unavailable to attend at the time a focus group was scheduled or had technical difficulties joining a focus group. Individual interviews (n=2) were conducted with these study participants to ensure that their perspectives were included. |
| Describe any insights gained from mixing or integrating methods | Strengths and limitations: pg. 20  Using a mixed method study design made it possible to use qualitative findings to clarify and elaborate on the quantitative results and to reveal additional information.  The inclusion of in-depth qualitative methods illustrates the richness and value of capturing subjective learning. |

O'Cathain, A., Murphy, E. and Nicholl, J. (2008) The quality of mixed methods studies in health services research. J Health Serv Res Policy;13: 92-98.
