# Supplementary material for: Embedding formal and experiential public and patient involvement training in a structured PhD programme: process and impact evaluation
Source: Res Involv Engagem. 2023 Nov 24;9:105. doi: 10.1186/s40900-023-00516-4 (PMC10668398; doi:10.1186/s40900-023-00516-4)
Supplement: Supplementary file 3 — Additional file 3. Impact Log Template [file 40900_2023_516_MOESM3_ESM.docx]

**Additional File 6**

**Impact log template**

| Date of PPI panel meeting (dd/mm/yy) |  | PhD scholar initials |  |
| --- | --- | --- | --- |
| Description of PPI activity |  | | |
| Summary of PPI contributors’ feedback and comments |  | | |
| Changes made to the research project |  | | |
| Perceived extent of changes (small / moderate / large) |  | | |
| Description of Impact |  | | |
| PhD scholars’ additional comments |  | | |
