# Supplementary material for: Embedding formal and experiential public and patient involvement training in a structured PhD programme: process and impact evaluation
Source: Res Involv Engagem. 2023 Nov 24;9:105. doi: 10.1186/s40900-023-00516-4 (PMC10668398; doi:10.1186/s40900-023-00516-4)
Supplement: Supplementary file 4 — Additional file 4. Guidance for Reporting Involvement of Patients and the Public 2 (GRIPP2) short form [file 40900_2023_516_MOESM4_ESM.docx]

**Additional File 4**

**Guidance for Reporting Involvement of Patients and the Public 2 (GRIPP2) short form**

| **Section and topic** | **Item** | **Reported on in Section and page no.** | **Text from paper** |
| --- | --- | --- | --- |
| Aim | Report the aim of PPI in the study | Background - under Aims of the study p. 3 | In addition to including PPI panel members in the evaluation as study participants, the study aimed to engage the PPI panel in planning the evaluation study and reviewing the plain language statement |
| Methods | Report a clear description of the methods used for PPI in the study | Methods - under Methods used for PPI in the evaluation, p. 5 | **Methods used for PPI in the evaluation**  The PPI panel were involved in the evaluation in several ways. At PPI panel meetings facilitated by PhD scholars, the PPI panel were consulted about the methods to be used for obtaining the perspectives of PPI panel members and questions to be asked. They decided to contribute their data using focus groups rather than through individual interviews. They identified the topics to be addressed by the evaluation and the questions used to guide their own focus groups. The findings from the analysis of their data were presented to the PPI panel members at a focus group to ensure that the results resonated with them and to give them an opportunity to reflect on the results. |
| Study results | Outcomes – Report the results of PPI in the study, including both positive and negative outcomes | Methods - under Methods used for PPI in the evaluation, p. 5  Results, p. 6 | **Methods used for PPI in the evaluation**  They [PPI panel] chose to contribute their data using focus groups rather than through individual interviews.  The evaluation findings were presented to the PPI panel who reported that they were accurate and resonated with their experiences and views. |
| Discussion and conclusions | Outcomes – Comment on the extent to which PPI influenced the study overall. Describe positive and negative effects | Discussion: p. 20 | During the planning of this evaluation, there was dialogue with the PPI panel, reflecting the CDA-MM programme’s view of PPI as a social practice of dialogue between researchers and the public,(1) which resulted in the use of focus groups to get the perspective of PPI members.  A positive effect of using this research method was that the PPI panel members got to collectively reflect on their experience of being involved in a PPI advisory panel. No negative effects were identified. |
| Reflections / critical perspective | Comment critically on the study, reflecting on the things that went well and those that did not, so others can learn from this experience | Strengths and limitations p. 20 | In consideration of the finite sample and dual roles of all study participants and to maintain a clear distinction between the research participant role and stakeholder role, it was decided not to engage in inter-stakeholder dialogue during data collection and analysis. The aim, in doing so, was to distinguish this research piece from usual PPI activities, where PhD scholars regularly engaged in dialogue and learning through reflection amongst stakeholders, including during the development of this evaluation study, and following regular PPI meetings. |

From: Staniszewska, S., Brett, J., Simera, I.*et al.* (2017) GRIPP2 reporting checklists: tools to improve reporting of patient and public involvement in research. *Res Involv Engagem* **3**, 13. <https://doi.org/10.1186/s40900-017-0062-2>

**References**

1. Russell J, Fudge N, Greenhalgh T. The impact of public involvement in health research: what are we measuring? Why are we measuring it? Should we stop measuring it? Research Involvement and Engagement. 2020;6(1):63.
